# A Municipal Milk Supply

**Published:** 1916-09-30

**Authors:** 


### A MUNICIPAL MILK SUPPLY.

The increase in the price of milk to the public —an increase which, it has been shown, is out of proportion to the increase in the cost of production, has inevitably revived proposals for the municipalisation of its supply. A few years ago, projects of this nature were under discussion, and with milk at a war price which, in the amount necessary for the health of child-life, renders it prohibitive to a large portion of our population, the problem of our milk supply again becomes acute. Public ownership is considered by some to be the best solution of the problem, and the arguments by which it is supported certainly deserve careful consideration by all who have the interests of public health at heart.

The public health is clearly concerned with both the price and the purity of milk. Milk being the indispensable food for child-life and of the highest hygienic value for people of all ages, it follows that in the interests of health it should be made as cheap as it is possible to be. Dearness means insufficiency for the poor. Competition, it is argued, makes for cheapness; but the argument hardly seems to apply to this commodity. The cost of distribution, as we have recently been reminded by the milk trade, forms an exceptionally heavy item in the cost to the consumer, and competition has given us the most wasteful method of distribution imaginable. To a street of fifty houses we shall find that competitive dairies send half a dozen men and vehicles, whereas with competition eliminated there would be a house-to-house delivery—as in the case of the post—at probably a third of the cost. Even more important than price is the question of purity. In theory, of course, the public are protected from these dangers by analysis and inspection. But as the reports of responsible medical officials have again and again shown, the protection is far from being complete. If it were made complete, the cost of the protection would probably only strengthen the demand of those who would take the milk supply out of private hands altogether. And further, as things are, the farms where the milk is produced are generally outside the jurisdiction of the inspecting authority of the population by which it is consumed. This consideration has led some advocates of a municipal supply to contend that municipalities must own not only the means of distribution, but also the means of production. Whereupon opponents of the proposal have exclaimed that the public acquisition of dairy farms " would involve a complete disorganisation of the farming industry of the country." Probably only experience would settle the question whether a municipality purchasing milk by contract from farmers could exercise an adequate control over its contractors. But its control would obviously be more effective than any it can now exercise. It must be admitted, however, that municipal ownership of the means of supply might very possibly have as its sequel the municipal ownership of the means of production. The probable effect of this upon the farming industry raises questions that are rather outside our province.

The magnitude of any measure of universal municipalisation is not to be denied. The capital outlay would probably be considerable. The property and services of only a small part of those at present engaged in the milk business could be absorbed, and dispossession without some kind of compensation for real loss is not to be thought of, even in the name of public health. Any large and heroic measure of municipalisation is, therefore, not likely to occur even under the provocation of unjustifiably high prices.

